# Sinus Node Dysfunction as the First Manifestation of Left Ventricular Noncompaction with Multiple Cardiac Abnormalities

**DOI:** 10.1016/s0972-6292(16)30651-9

**Published:** 2013-08-01

**Authors:** Baris Gungor, Ahmet T Alper, Ahmet Celebi, Osman Bolca

**Affiliations:** 1 Department of Cardiology, Siyami Ersek Thoracic and Cardiovascular Surgery Center, Training and Research Hospital, Istanbul, Turkey; 2 Department of Pediatric Cardiology, Siyami Ersek Thoracic and Cardiovascular Surgery Center, Training and Research Hospital, Istanbul, Turkey

**Keywords:** Cardiomyopathy, congenital heart disease, sinus node dysfunction

## Abstract

Left ventricular noncompaction (LVNC) is a genetically heterogenous form of cardiomyopathy which may remain undiagnosed till adulthood due to the late presentation of typical symptoms such as dyspnea, congestion, ventricular arrhythmias and thromboembolism. Symptomatic bradycardia secondary to persistent sinus node dysfunction is very rare. Coexistent cardiac defects are common in children however in adults the disease is usually in isolated form. Here, we present a case of twenty-three year-old female LVNC patient with patent ductus arteriosus, bicuspid aortic valve and persistent sinus node dysfunction who presented with dizziness as the first manifestation of the disease.

## Introduction

Left ventricular noncompaction (LVNC) is a myocardial disorder characterized by excessive trabeculations and deep recesses that communicate with the ventricular cavity. Concomitant heart defects, mostly ventricular septal defects are encountered in these patients and are accepted as a result of ventricular maldevelopment during embryologic life. The major manifestations of the disease are heart failure, thromboembolism and ventricular arrhythmias whereas symptomatic bradycardia is very rare. [1,2]. Here, we report a case of LVNC with multiple cardiac abnormalities in an adult patient whose first symptom was dizziness secondary to sinus node dysfunction.

## Case Report

**Figure 1:**
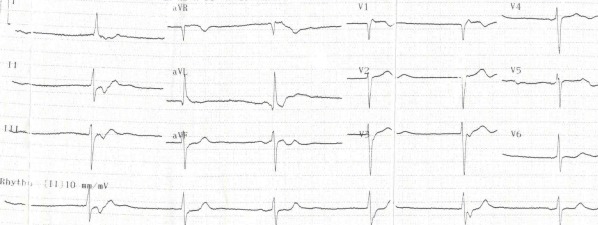
12-Lead electrocardiogram showing junctional rhythm.

**Figure 2:**
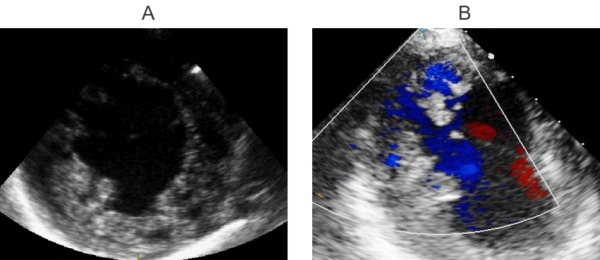
Transthoracic echocardiography showing left ventricular noncompaction of A) lateral wall at parasternal short axis view B) apical and apicolateral wall at apical 4-chamber view with noncompacted:compacted myocardium ratio of more than 2. Color doppler imaging revealed communication between myocardial recesses and left ventricular cavity.

**Figure 3:**
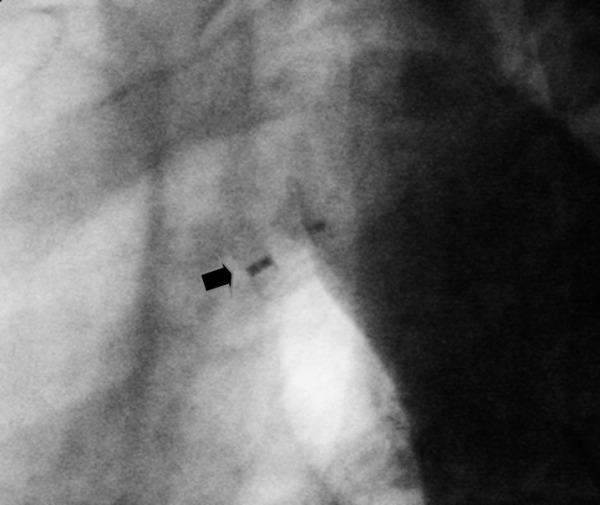
Aortogram showing total occlusion of ductus arteriosus after implantation of ductus occluder device (arrow).

A 23-year-old female patient admitted to our emergency department with a complaint of dizziness which started within 12 hours. On physical examination her skin was pale and diaphoretic, her pulse was regular but bradycardic with a rate of 40 beats/min. Her arterial blood pressure was measured 110/70 mmHg. On auscultation, a continuous murmur which was loudest at the left upper chest was determined. The remainder of the physical examination was normal. Her medical and family history was unremarkable. A 12-lead electrocardiogram revealed QRS complexes of 100 msec width with regular R-R intervals without any preceding p waves (Figure 1). In laboratory examination, biochemical parameters including electrolyte levels and thyroid function were within normal limits. Transthoracic echocardiography revealed bicuspid aortic valve, mildly depressed left ventricular (LV) systolic function, hypertrabeculation of LV myocardium involving apical and midventricular portions of lateral wall, deep intertrabecular recesses communicating with the LV cavity with a noncompacted: compacted myocardium ratio of 2.5 (Figure 2). In parasternal short axis view color doppler examination demonstrated a turbulant flow from aorta to the pulmonary artery compatible with patent ductus arteriosus (PDA). As the shunt was hemodynamically significant percutaneous closure of the ductus arteriosus by Cardi-O-Fix duct occluder (Starway Medical Technology Inc. Beijing, China) was performed (Figure 3). Electrophysiologic study (EPS) showed that sinus node was non-functional and electrical activity originated near the atrioventricular node. Programmed electrical stimulation did not establish any sustained ventricular arrhythmias. A dual pacemaker without cardioverter properties was implanted and the patient was discharged uneventfully. At 3 and 6 months follow-up the patient was asymptomatic and pacemaker analysis showed total loss of sinus node electrical activity.

## Discussion

Left ventricular noncompaction is accepted as a genetically heterogenous form of cardiomyopathy and was initially defined in pediatric population with congenital syndromes [3]. Heart failure symptoms including fatigue, dyspnea and palpitations are the most common presenting symptoms. Dizziness as the first symptom of LVNC is relatively rare and mostly occurs secondary to tachyarrhythmias.

Arrhythmias are common in LVNC and sudden cardiac death is the major cause of mortality which is reported to be 4% per year in isolated LVNC cases [4]. Formation of reentrant circuits thru intertrabecular recesses is accepted as the mechanism of ventricular arrhythmias.

In addition, pre-excitation syndromes, atrioventricular and interventricular conduction disturbances may be encountered in LVNC patients [5,6]. Steffel et al. reported the frequency of LBBB and PR interval prolongation as 19% and 15% respectively in a cohort of 78 LVNC cases with a mean LVEF of 40% [6]. Ozkutlu et al reported a case of sustained bradycardia in a fetus with isolated LVNC which resulted in junctional rhythm on ECG after delivery [7]. Caliskan et al. reported a 23 year old male patient with symptomatic sinus node dysfunction with moderate aortic regurgitation, severe LV dilatation and LVNC [8]. However, the authors have also considered the possibility of pseudo-LVNC pattern secondary to chronic volume overload and severe bradycardia. In our case, we presume that LVNC is the primary abnormality which resulted in sinus node dysfunction. Ichida et al. observed interstitial fibrosis and subendocardial fibroelastosis in endomyocardial biopsy specimens from LVNC cases and noted that progressive endocardial fibrosis may lead to conduction abnormalities [5]. It can be hypothesized that advanced subendocardial fibrosis may impair atrial conduction and in severe cases may result in deterioration of sinus node function.

In adult population, LVNC is mostly in isolated form. Zavelata et al. reported coexistence of other heart defects with a proportion of 26% in 23 adult LVNC cases including 2 cases of PDA and a case of bicuspid aortic valve [9]. In addition, there are two case reports of LVNC accompanied with PDA [10,11]. In pediatric population, PDA incidence in noncompaction cases has been reported as 14% [12]. In our case, PDA and bicuspid aortic valve were asymptomatic and diagnosed incidentally during cardiac examination. PDA was percutaneously closed and as bicuspid aortic valve did not cause significant stenosis or regurgitation, any intervention was not performed. Our case implies that, all of the LVNC cases including asymptomatic ones should be investigated in detail for any coexistent cardiac abnormality including bicuspid aortic valve and PDA.

To date, intracardiac cardioverter defibrillator (ICD) implantation is the most promising therapy in LVNC patients with ventricular arrhythmias. Guidelines recommend ICD implantation in ventricular noncompaction cases who are survivors of sudden cardiac death; who have spontaneous sustained ventricular tachycardia whether hemodynamically unstable or stable; who have a left ventricular ejection fraction less than or equal to 35%. In our case, as a sustained ventricular arrhythmia was not detected and left ventricular systolic function was mildly depressed, we implanted a dual pacemaker without cardioverter properties. During the follow-up ventricular arrhythmias were not detected and the patient was asymptomatic.

In conclusion, our case shows that persistent sinus node dysfunction may be the first symptom in LVNC cases. Considering LVNC as a congenital heart disease, accompanying heart defects including PDA and bicuspid aortic valve should be investigated in every patient with different imaging modalities when necessary.
